# The Teeth during Pregnancy

**Published:** 1893-05

**Authors:** 


ARTICLE II.

### THE TEETH DURING PREGNANCY.

The belief is very common among dentists that the teeth of women suffer during gestation, because of the needs of the growing organism. That in some way the teeth of the mothers are robbed of their lime salts, to supply the demands of the fœtus. We cannot but think this a grave error.

For a long time the late Dr. John Allen annually presented a paper before some dental body, devoted to an exposition of the wrong done by the millers in bolting flour. He reasoned after this manner: The teeth of Americans are notoriously bad. Those of the savage tribes are as notoriously good. The gluten of grain lies next the bran. The Americans bolt this out that they may get flour that will make white bread. Savage tribes do not do this. The bad teeth of our people are due to a lack of this bone-making material in their food. Hence the millers of the country are responsible for our bad teeth.

This was ingenious, but it contained a number of serious errors. Perhaps the most important of these lies in the second predicate, for it is not true that aboriginal or savage people have good teeth. On the contrary, some barbarous tribes have worse teeth than Americans.

Again, while it is true that the gluten of our cereal grains is especially rich in the phosphates, it is not true that fine flour is without sufficient for all the needs of man. The following computation has been made: If rice flour, which contains as little of the phosphates as any other common food, were the sole nutrition of a pregnant woman, and if she consumed barely enough to maintain a healthy existence, she might obtain from that alone double the amount that would be needed for herself and the growing child. It is well known that women always excrete phosphates during gestation. Fine wheat flour contains more of the bone-making elements than does rice flour; hence it cannot be that our bad teeth are due to lack of the proper material in our food.

The corollary to the hypothesis so long held concerning the nutrition of the teeth, naturally was that the lack of lime salts in the food must be artificially supplied, and hence the many preparations of calcium that were formerly urged upon the people. The truth is, that under no circumstances can the animal organize the inorganic. The function rests solely with the Vegetable Kingdom. All of the inorganic elements of the body must be derived from organic sources. Hence it is the wildest kind of vagary to prescribe any inorganic material for nutrient purposes. It cannot be built into the tissues, and must invariably be excreted if taken into the animal system. No inorganic matter was ever yet accepted and built up by any animal organism. Such elements may have their uses in the system, but it must always be as medicines. Their presence may induce structural changes through their medicinal action, but they themselves are never used for trophic purposes. It follows, then, that the giving of any form of the phosphates, in the expectation that it will be used in nutrition, is the result of ignorance of physiological law.

To go back, then, to the cause of the decay of the teeth during pregnancy. It cannot be due to the lack of the proper ingredients in the food, provided the mother has sufficient of that which is wholesome. If the nutrient processes are in a healthy state, they will find plenty of material out of which to build bones and teeth. Besides, if there were a scarcity of the lime salts, why should it manifest itself in the teeth alone. Or, being felt, why should not the number of teeth be diminished instead of the quality? Why might it not be that there are but four toes, or fingers, or why should not some bone be deficient in length, or size, if the material proved insufficient?

But that the teeth of the mother should be robbed of their lime salts to help out the fœtus, seems to us the most absurd of theories. There could be but one way in which the tooth could thus be depleted. There must, in that case, be a solution of the salts and their taking up by a system of absorbents. But there are no such absorbent vessels in the tooth. It is true that under certain circumstances a tooth may be absorbed, but when this is done the whole of the tissue goes; it is taken up into the system by absorbent vessels, whence it is excreted. To secure this result a special system of cells is developed—the osteoclasts—and these do the work. There are no such, or any other, absorbent cells in the hard tissue of the tooth, and hence there cannot be any such absorption.

There is no doubt that the character of the tooth tissue changes with its nutrition, or mal-nutrition, but not through any such process as that sometimes claimed. No, the expectant mother neglects her teeth. She has sufficient upon her mind to make her forget the tooth-brush She goes to bed with her attention fixed on other matters, and her teeth are neglected. She awakes in the morning, perhaps with nausea, and she is in no mood to brush her teeth. Besides, her appetites are apt to be capricious, and she deranges her stomach by improper food, or possibly it sympathizes with the gravid uterus. The secretions are perhaps changed, and these morbid conditions add to the trouble. Her nutrition is interfered with, and the teeth are not properly nourished. All these things produce their natural result in caries of the teeth, and the etiology is traced to her condition, and that is made the primary cause, whereas it is only secondary.

Let the mother keep up the hygienic precautions usual with her under other circumstances; let her clean her teeth often and carefully; let her food be sufficient and wholesome, and her nutritive processes be in good condition, and there is no reason why her teeth should especially decay during pregnancy, or why their nutrition should in any way be interfered with.—EDITORIAL in *Dental Practitioner and Advertiser.*

---

ARTICLE III.

